# Galectin-3 and HFpEF: Clarifying an Emerging Relationship

**DOI:** 10.2174/1573403X19666230320165821

**Published:** 2023-07-17

**Authors:** Basil M. Baccouche, Emmajane Rhodenhiser

**Affiliations:** 1 School of Medicine, Stanford University, Stanford, California, USA;; 2 Department of Nanoscience, Brown University, Providence, Rhode Island, USA

**Keywords:** HFpEF, heart failure, biomarker, galectin-3, cardiovascular disease, risk factor

## Abstract

**Introduction:**

HFpEF is one of the leading causes of death whose burden is estimated to expand in the coming decades. This paper examines the relationship between circulating levels of galectin-3, an emerging risk factor for cardiovascular disease, and the clinical diagnosis of HFpEF.

**Methods:**

The authors reviewed peer-reviewed literature and 18 studies met the inclusion criteria. Study characteristics, study outcome definitions, assay characteristics, main findings, and measures of association were tabulated and summarized.

**Results:**

Five studies found significant associations between galectin-3 and HFpEF diagnosis compared to healthy controls, and one did not. Five studies found significant associations between galectin-3 concentration in circulation and severity of diastolic dysfunction. Three studies found a statistically significant association between circulating galectin-3 and all-cause mortality or rehospitalization. Two studies found levels of circulating galectin-3 to be a statistically significant predictor of later HFpEF onset. Finally, two studies examined whether galectin-3 was associated with incident HFpEF, one found a significant association and the other did not.

**Conclusion:**

Given the paucity of effective therapeutics for HFpEF, galectin-3 shows promise as a possible HFpEF-linked biomarker that may, with further study, inform and predict treatment course to reduce morbidity and mortality.

## 1 INTRODUCTION

Cardiovascular disease (CVD) is the number-one cause of death in the United States [1-18]. Heart failure with preserved ejection fraction (HFpEF) is a type of CVD in which heart failure symptoms are observed but the fraction of blood ejected to systemic circulation is conserved, likely due to impaired ventricular filling [19-24]. The burden of HFpEF has increased in the last decade, currently representing a majority of all cases of HF [25, 26]. The global burden is anticipated to increase as well in the coming decades [27]. Despite this alarming trend, there are still no effective therapies for HFpEF [28].

Galectin-3, classified in the galectin family, is a protein that has been shown to be causally linked to pathophysiological cardiovascular processes, including atherosclerosis, fibrosis, and heart failure [29]. Galectin-3 has been presented in the literature as a novel biomarker for cardiac disease diagnosis, and a recent meta-analysis has shown that levels of circulating galectin-3 are associated with incident heart failure [30, 31].

As the global burden of heart disease continues to grow, so too does the importance of advancing preventative tools like biomarker-based risk prediction models. An incomplete understanding of the molecular and pathophysiological pathways underlying the development of HFpEF is one of many factors limiting the development of an effective HFpEF therapy.

The aim of this article is to interrogate and clarify the relationship between HFpEF diagnosis and concentration of circulating galectin-3 through a rigorous, reproducible review of the published literature associating HFpEF-related-endpoints and galectin-3. The authors, to the best of their knowledge, have not identified any review examining this specific relationship and thus hope that this synthesis of findings will prove useful for the investigator interested in galectin-3 as a potential biomarker for HFpEF.

## 2 METHODS

The Sciome Workbench for Interactive computer-Facilitated Text-mining (SWIFT)-Review, was used to perform a review examining the association between levels of galectin-3, a circulating biomarker, and incidence of heart failure with preserved ejection fraction (HFpEF) [29, 32, 33]. SWIFT is an efficient tool that uses statistical text mining to make it easier to manually screen results. Table **1** outlines the search terms that were processed within the National Library of Medicine’s MEDLINE database: [(galectin-3 OR gal-3) AND (HFpEF)], with zero restriction settings applied to the search tool. Because of the low number of total results, automated screening results were double-checked *via* manual screening by the authors. Study inclusion criteria are shown in Table **1**, and a PRISMA-compatible flow chart is included in Fig. (**1**) for transparency and reproducibility [34].

All intra-study data were extracted manually by the authors.

## 3 RESULTS

### 3.1 Characteristics

18 studies met the inclusion criteria outlined in Table **1**. Amongst the 18 qualifying studies, six were case-control studies, seven were prospective cohort studies, four were cross-sectional, and one was a retrospective cohort study. The sample size across all 18 studies ranged from 62 and 22,756, and the average participant age was between 55.9 and 75 years. The percentage of female participants ranged from 11.50% to 53.30%, with most studies nearly 50% female. 12 of the 18 study populations included diabetics. Studies were all community-based, and were conducted in China, Europe, India, Russia, Taiwan, and the United States. Table **2** summarizes the study characteristics.

### 3.2 Outcomes

All 18 studies included the clinical diagnosis of HFpEF as a variable of interest. Outcomes were ascertained using validated standardized criteria, except for two studies [1, 6] that used HFpEF as diagnosed internally by a cardiologist(s). All studies used echocardiographic data in combination with other clinical measurements to confirm the diagnosis of HFpEF. Eight studies indicated the follow-up period, ranging from 0.5 months to 144 months. Study-specific HFpEF definitions and outcome ascertainment methods are shown in Table **S1**.

To measure galectin-3 concentration in circulation, three studies [6, 7, 15] used Abbot Laboratories’ Architect System, one study [12] used a human galectin-3 assay kit, another [2] used a Quantikine USA kit, and the rest all used enzyme-linked immunosorbent assays (ELISAs) from various manufacturers. Detailed galectin-3 measurement information, including storage temperature and storage duration (where provided), is shown in Table **S2**.

### 3.3 Main Findings

An overview of the main findings from each study is shown in Table **3** and described herewith. Outcome definitions were heterogenous across studies and thus published data were not suitable for meta-analysis. Measures of association (where provided) and *p*-values, as well as the comparisons under investigation in each study, are shown in Table **4**. All studies provided at least one measure of statistical significance (measure of association, *p*-value, or both).

Galectin-3 levels were compared with one of five endpoints: levels in healthy controls, the severity of diastolic dysfunction, all-cause mortality or rehospitalization, development of HFpEF, and prediction of HFpEF diagnosis. Of the total 18 included studies, six investigated the relationship between levels of circulating galectin-3 and HFpEF diagnosis compared to healthy controls; five [1-5] of those six found statistically significant associations, and one [6] did not. Five studies probed the relationship between galectin-3 concentrations and the severity of diastolic dysfunction and all five [7-11] found statistically significant associations. Three studies examined the relationship between levels of circulating galectin-3 and all-cause mortality or rehospitalization, and all three [12-14] found statistically significant associations. Two studies [15, 16] found levels of circulating galectin-3 to be a statistically significant predictor of later HFpEF onset. Finally, two studies examined whether levels of circulating galectin-3 were associated with current HFpEF; one [17] found an association that did not meet the threshold of statistical significance, and the other [18] found a significant association.

## 4 DISCUSSION

### 4.1 Findings & Implications

This review demonstrates a consistent pattern of statistically significant association between circulating galectin-3 levels and respective HFpEF endpoints. The variance in study methodology prohibits meta-analysis, but the synthesis of data herein nevertheless provides valuable insight into the studied relationship between galectin-3 and HFpEF. Endpoints included incident HFpEF, the severity of diastolic dysfunction as assessed by echocardiography or other clinical measurements, the severity of myocardial fibrosis, and all-cause mortality/rehospitalization due to HFpEF. In 16 of the 18 studies, elevated galectin-3 levels were found to be significantly associated with HFpEF patients *vs.* controls, distinguished between HFpEF and other HF subtypes, or correlated positively with other well-established markers of cardiac dysfunction.

There is an immense, rapidly-growing burden of HFpEF, a well-documented lack of effective treatment, a relative paucity of studies investigating this promising relationship, and a high heterogeneity in HFpEF endpoint across studies that investigate this relationship (limiting meta-analysis). Therefore, the authors suggest that the emerging biomarker galectin-3 - which has been implicated in the pathogenesis of cardiovascular remodeling [30] - should be rigorously interrogated in a large cohort using metrics of risk prediction for HFpEF.

Galectin-3 is widely expressed throughout the body and elevated concentration is implicated in kidney disease, heart disease, and liver disease. The multifunctional role of galectin-3 makes it challenging to isolate, as depending on its location in the cell, it has been observed to play roles in cell survival, gene transcription, or cell-cell interactions [30]. Animal models with increased galectin-3, knockout galectin-3, or pharmacological inhibition of galectin-3 have suggested mechanisms of action by which galectin-3 may influence cardiac fibrosis [35]. These studies show that injury, hypertension or aldosteronism can increase galectin-3, which may promote cardiac remodeling by depositing collagen and inducing fibroblast proliferation after being expressed in active macrophages and cardiomyocytes. Increased collagen deposits can lead to myocardial fibrogenesis, and subsequent cardiac remodeling [35].

HFpEF is a complex physiological phenomenon and is unlikely to be univariately associated with galectin-3 (or any single biomarker). It is certainly the case that a concert of contributing factors is responsible for the diverse physiological dysfunctions and subsequent symptoms associated with this debilitating disease. However, in seeking to unravel the question of how and when patients develop HFpEF, this review makes a case for galectin-3’s inclusion among potential biomarkers.

Only two of the 18 studies found borderline or statistically insignificant associations, and none of the studies reported an inverse association between galectin-3 and the HFpEF endpoint. Of the two studies which did not report a statistically significant association, one study [17], while high in sample size, pooled hazard ratios across four studies. One of those four studies (MESA) did not have data for inclusion in this pooled measure of association. Two of the remaining three studies reported a significant association between galectin-3 and incident HFpEF, and the third found no association; when pooled, no association prevailed. Finally, they limited the inclusion of HFpEF diagnoses in the pooled dataset only to individuals presenting with HF and undergoing left ventricular function assessment, resulting in 30% of cases with unclassified HF [17]. As a result, although this study had an impressive sample size, it is recommended that the relationship between incident HFpEF and galectin-3 in particular be interpreted with caution. The other study which did not report a statistically significant association [6] reported a *p*-value of 0.06, just above the predefined significance threshold; an argument may be made, given its borderline *p*-value and modest sample size (n = 115), for its potential inclusion within the domain of clinical significance.

### 4.2 Study Limitations

The authors did not review pre-prints journals not indexed in MEDLINE. Galectin-3 is a biomarker associated with a range of cardiovascular dysfunctions and is therefore likely to be limited in utility to use in combination with other diagnostic tools to determine wall thickness, symptomatology, or ejection fraction. The relative lack of studies investigating this association means that the few studies that are available investigate disparate HFpEF endpoints and so are not suitable for pooled meta-analysis, even by HFpEF endpoint subgroup (as sample sizes are small). In addition, each of the 18 included studies is subject to its own methodological limitations, shown in Table **S4**. The most common limitations include a lack of other biomarkers as covariates, retrospective methods, and small sample sizes.

## CONCLUSION

In a review of 18 studies examining relationships between circulating galectin-3 and HFpEF diagnosis, diastolic dysfunction severity, incident HFpEF, or all-cause mortality/rehospitalization, 16 found statistically significant associations and 2 found borderline non-significant associations that are nevertheless of clinical interest. Given the scarcity of effective therapeutics for HFpEF, galectin-3 shows promise as a possible HFpEF-linked biomarker deserving of further study.

## Figures and Tables

**Fig. (1):**
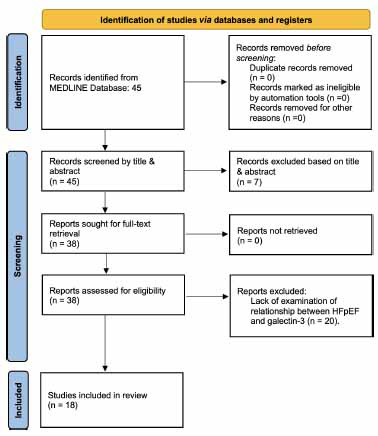
Reproducible, PRISMA-compatible review workflow [34].

**Table 1:** Study inclusion criteria.

**Inclusion Criteria**
• Human subject research • English language • Full-text freely available to the University of Cambridge • HFpEF clearly defined and diagnosed • Includes the exposure galectin-3 • Includes the outcome HFpEF diagnosis • Primary research

**Table 2:** Study characteristics.

**Authors/Refs.**	**Study Design**	**Study Period**	**Geographic Location(s)**	**Population Source(s)**	**Sample Size**	**Mean Age**	**%** **Female**	**% Diabetics**
Yin *et al.*, 2014 [3]	Case Control	2013	China	Community-based	78	71.86	11.50%	42.31%
Wu *et al.*, 2015 [8]	Cross-Sectional	N/A	Taiwan	Community-based	176	68.23	38.07%	N/A
Yu *et al.*, 2015 [13]	Prospective Cohort	2010-2011	China	Community-based	261	70.02	50.96%	N/A
Edelmann *et al.*, 2015 [14]	Prospective Cohort	2007-2011	Germany & Austria	Community-based (Aldo-DHF)	415	67.00	52.30%	16.60%
Berezin *et al.*, 2016 [16]	Case Control	2012-2015	Ukraine	Community-based	199	55.90	47.70%	19.10%
Beltrami *et al.*, 2016 [9]	Prospective Cohort	2012-2015	Italy	Community-based	98	74.84	51.02%	34.69%
Polat *et al.*, 2016 [4]	Case Control	2013-2014	Turkey	Community-based	82	58.61	46.34%	34.15%
de Boer *et al.*, 2018 [17]	Prospective Cohort	CHS: 1989-1990,1992-1993 FHS: 1995-1998 MESA:2000-2002 PREVEND:1997-1998	United States	Community-based (FHS, CHS, PREVEND, and MESA)	22756	60.00	53.12%	10.00%
Wu *et al.*, 2018 [10]	Cross Sectional	2011-2015	Taiwan	Community-based	77	67.79	55.86%	N/A
Cui *et al.*, 2018 [12]	Case Control	2014-2016	China	Community-based	247	71.93	54.70%	30.77%
Ansari *et al.*, 2018 [7]	Prospective Cohort	2014-2016	Germany	Community-based	70	65.00	49.00%	24.00%
Lebedev *et al.*, 2020 [5]	Case Control	N/A	Russia	Community-based	62	59.29	43.55%	100.00%
Merino-Merino *et al.*, 2020 [6]	Cross Sectional	2015-2017	Spain	Community-based	115	62.78	30.43%	13.91%
Pecherina *et al.*, 2020 [11]	Prospective Cohort	2015	Russia	Community-based	254	60.64	30.71%	16.14%
Mitic *et al.*, 2020 [2]	Cross Sectional	2018	Serbia	Community-based	112	N/A	N/A	N/A
Kanukurti *et al.*, 2020 [1]	Case Control	N/A	India	Community-based	83	57.25	N/A	N/A
Watson *et al.*, 2021 [15]	Retrospective Cohort	2009-2011	Ireland	Community-based (STOP-HF)	90	75.00	53.30%	N/A
Trippel *et al.*, 2021 [18]	Prospective Cohort	2004-2016	Germany	Community-based	1386	67.00	50.90%	26.60%

**Table 3:** Main findings of each study, as reported in the body of the text (and edited for concision). Insignificant results are provided in *italics* for clarity.

**Study Authors**	**Main Findings**
Yin *et al.*, 2014	Galectin-3 levels were significantly higher in HFpEF patients compared to controls.
Wu *et al.*, 2015	Tissue and plasma galectin-3 were significantly correlated with the degree of diastolic dysfunction and severity of myocardial fibrosis.
Yu *et al.*, 2015	Galectin-3 levels were significantly higher in CHD HF patients, and galectin-3 was an independent predictor of both all-cause mortality and hospital readmittance.
Edelmann *et al.*, 2015	Galectin-3 levels are higher in HFpEF patients, and galectin-3 is associated with hospitalization in HFpEF patients.
Berezin *et al.*, 2016	Galectin-3 is an independent predictor of HFpEF.
Beltrami *et al.*, 2016	Galectin-3 levels are associated with diastolic dysfunction severity and LV stiffness in HFpEF patients.
Polat *et al.*, 2016	Galectin-3 was elevated in HFpEF patients compared to controls, and was correlated with NT-proBNP, left atrial volume index, LV mass index, and E/E’.
de Boer *et al.*, 2018	Galectin-3 was not associated with incident HFpEF.
Wu *et al.*, 2018	Galectin-3 was associated with fibrosis in HFpEF patients.
Cui *et al.*, 2018	Galectin-3 levels distinguished HFpEF patients from controls and were correlated with an increased risk of endpoint events in HFpEF patients.
Ansari *et al.*, 2018	Galectin-3 was associated with HFpEF diagnosis by echocardiogram, disease course, and diastolic dysfunction severity.
Lebedev *et al.*, 2020	Galectin-3 levels were significantly higher in HFpEF and HFmrEF patients compared to controls.
Merino-Merino *et al.*, 2020	Galectin-3 was not associated with HFpEF patients compared to controls.
Pecherina *et al.*, 2020	Galectin-3 levels were higher in HFpEF compared to HFrEF patients and were associated with diastolic dysfunction severity.
Mitic *et al.*, 2020	Galectin-3 was associated with diastolic dysfunction severity in HFpEF patients compared to controls.
Kanukurti *et al.*, 2020	Galectin-3 was elevated in HFpEF patients compared to controls and was more sensitive in diagnosing HFpEF than NT-proBNP. higher in HFpEF patients and positively correlated with NT-ProBNP and lipid parameters.
Watson *et al.*, 2021	Galectin-3 levels predicted incident HFpEF.
Trippel *et al.*, 2021	Galectin-3 levels were associated with incident HFpEF, hospitalization, and mortality at ten years follow-up.

**Abbreviations:** Area Under the Curve (AUC); B-type natriuretic peptide (BNP); Congenital Heart Defects (CDH); Heart Failure (HF); Heart Failure with Preserved Ejection Fraction (HFpEF); Heart Failure with Reduced Ejection Fraction (HFrEF); High Sensitivity Troponin-I (hsTroponin-I); Left Ventricular (LV); Left Ventricular Ejection Fraction (LVEF); NT-proB-type Natriuretic Peptide (NT-proBNP); Receiver operating characteristic (ROC).

**Table 4:** Measures of association (if provided) of included studies. If only one measure of association was provided, it was considered “further-adjusted”. *p*>0.05 is provided in *italics* for clarity. Table S3 provides minimally adjusted HR, when provided.

**Study ** **Authors**	**Further Adjusted HR [95% CI],** ***P*-value**	**Comparison**	**Covariates Used in Further Adjusted Model**
Yin *et al.*, 2014	N/A, *p* = 0.000	Galectin-3 and HFpEF diagnosis *vs*. controls (no HF).	N/A
Wu *et al.*, 2015	N/A, *p <* 0.001	Galectin-3 and severity of diastolic dysfunction.	Age, diabetes, gender, LV mass index, plasma NT-proBNP and prescribed drugs.
Yu *et al.*, 2015	*RR: 1.231, 95% [1.066-1.442] *p* = 0.005	Galectin-3 and all-cause mortality and rehospitalization.	N/A
Edelmann *et al.*, 2015	3.319 [1.214-9.07] *p* = 0.019	Galectin-3 and all-cause death or hospitalization.	Peak VO2, six min walk distance, and short form 36 physical function.
Berezin *et al.*, 2016	1.08 [1.03-1.12] *p* = 0.002	Galectin-3 and prediction of HFpEF diagnosis.	Diabetes type 2, mellitus, obesity, previous myocardial infarction.
Beltrami *et al.*, 2016	19.62 [2.39-60.89] *p* = 0.006	Galectin-3 and severity of diastolic dysfunction.	CKD, diabetes, dyslipidemia, hypertension, smoker.
Polat *et al.*, 2016	N/A, *p* < 0.0001	Galectin-3 and HFpEF diagnosis *vs*. controls (no HF).	N/A
de Boer *et al.*, 2018	1.02 [0.93-1.12] *p = 0.13*	Galectin-3 and incident HFpEF.	Age, BMI, diabetes, hypertension treatment, L ventricular hypertrophy, L bundle branch block, previous myocardial infarction, race/ethnicity, sex, systolic blood pressure, smoking.
Wu *et al.*, 2018	*OR: 1.05 [1.02 - 1.0^9^] *p* = 0.005	Galectin-3 and severity of diastolic dysfunction.	Diabetes, Endothelin-1, Heart failure, MMP-2, NT-proBNP, TIMP2.
Cui *et al.*, 2018	2.33 [1.72–2.94] *p* = 0.009	Galectin-3 and all-cause death or hospitalization.	Age, aldosterone receptor antagonist, b-blockers treatment, coronary artery disease, diastolic blood pressure, eGFR levels, hypertension, LDL cholesterol levels, LVEF, NT-proBNP levels, NYHA grade, sex, systolic blood pressure.
Ansari *et al.*, 2018	*OR: 6.19 [1.489–25.744] *p* = 0.012	Galectin-3 and severity of diastolic dysfunction.	Age, gender, NT-proBNP, and serum creatinine.
Lebedev *et al.*, 2020	N/A, *p* = 0.01	Galectin-3 and HFpEF diagnosis *vs*. controls (no HF).	N/A
Merino-Merino *et al.*, 2020	N/A, *p = 0.06*	Galectin-3 and HFpEF diagnosis *vs*. controls (no HF).	Age, arterial hypertension, diabetes, obesity, and sex.
Pecherina *et al.*, 2020	N/A, *p* < 0.0001	Galectin-3 and severity of diastolic dysfunction.	N/A
Mitic *et al.*, 2020	N/A, *p* < 0.001	Galectin-3 and HFpEF diagnosis *vs*. controls (no HF).	Age, BMI, GDF-15, sST2, and syndecan-1.
Kanukurti *et al.*, 2020	N/A, *p* < 0.0001	Galectin-3 and HFpEF diagnosis *vs*. controls (no HF).	Age, comorbidities, sex, and troponin.
Watson *et al.*, 2021	*OR: 1.17 [1.02-1.^34^] *p* = 0.027	Galectin-3 and prediction of HFpEF diagnosis.	Age, sex, levels of: hsTropI, IL6, and ln(BNP) and sST2.
Trippel *et al.*, 2021	*OR: 1.77 [1.14-2.74] *p* = 0.010	Galectin-3 and incident HFpEF.	Age, BMI, diabetes mellitus, hypertension, kidney function, and sex.

**Note:** *These studies provided an odds ratio (OR) or risk ratio (RR) as a measure of association instead of a hazard ratio.

## Data Availability

All the data-supportive information is provided within the article.

## LIST OF ABBREVIATION

ACE: Angiotensin-converting Enzyme
BMI: Body Mass Index
BNP: B-type Natriuretic Peptide
CI: Confidence Interval
CKD: Chronic Kidney Disease
eGFR: Estimated Glomerular Filtration Rate
GDF-15: Growth/Differentiation Factor-15
HR: Hazard Ration
HF: Heart Failure
HFmrEF: Heart Failure with Midrange Ejection Fraction
HFpEF: Heart Failure with Preserved Ejection Fraction
hsTropI: High Sensitivity Troponin I
IL6: Interleukin-6
LV: Left Ventricular
LDL: Low Density Lipoprotein
MMP-2: Matrix Metalloproteinase-2
NYHA: New York Heart Association
NT-proBNP: N-terminal-pro hormone B-type Natriuretic Peptide
RR: Risk Ration
sST2: Soluble Suppression of Tumorigenicity-2
SD: Standard Deviation
TIMP2: Tissue Inhibitor of Metalloproteinases-2

## STANDARDS OF REPORTING

PRISMA guidelines and methodology were followed.
