# Supplementary material for: Galectin-3 and HFpEF: Clarifying an Emerging Relationship
Source: Curr Cardiol Rev. 2023 Jul 17;19(5):e200323214796. doi: 10.2174/1573403X19666230320165821 (PMC10518880; doi:10.2174/1573403X19666230320165821)
Supplement: Supplementary file 1 [file CCR-19-E200323214796_SD1.pdf]

Supplementary Material

Galectin-3 and HFpEF: Clarifying an Emerging Relationship

Basil M. Baccouche[1] and Emmajane Rhodenhiser[2,*]

[1]*Stanford University School of Medicine, Stanford, California, USA;* [2]*Brown University, Providence, Rhode Island, USA*

Table S1. Study outcomes.

Authors	Defining Outcome of Interest (HFpEF)	Follow-Up Length in Years	Outcome Collection	Outcome Ascertainment
Yin et al., 2014	(1) LVEF > 45% (2) standard size of the left ventricular cavity, and (3) left ventricular diastolic dysfunction as confirmed by echocardiography. Functional classification per NYHA.	N/A	Echocardiography, medical records, plasma samples.	NYHA
Wu et al., 2015	HF as previously diagnosed site. HFpEF severity as determined by Doppler imaging (E/Em) and CMRI.	N/A	Cardiac magnetic resonance imaging, doppler imaging, echocardiography, hemody-namic data, medical records, plasma samples.	Framingham criteria
Yu et al., 2015	HF as diagnosed per ESC 2008, and functional classification as assigned according to NYHA.	12 months	Angiography, clinical as-sessment, electrocardiog-raphy, telephone interviews.	ESC, NYHA
Edelmann et al., 2015	HFpEF as previously defined by the Aldo-DHF study. There were four criteria for participation: (1) NYHA class II or III (2) LVEF ≥50%, (3) Grade ≥ I diastolic dysfunction 22 or current AF, per echocardiogram and (4) peak VO2≤ 25 mL/kg/min.	12 months	Echocardiography, medical records, plasma samples.	NYHA
Berezin et al., 2016	Chronic HF as per contemporary clinical criteria. HFrEF (LVEF ≤ 45%) and HFpEF (LVEF ≥ 50%).	N/A	Echocardiography, doppler Imaging, medical records, serum samples.	ESC
Beltrami et al., 2016	All patients had (1) new onset or exacerbated AHF, (2) acute decompensated HF per signs and symptoms. HFrHF (EF below 50%) or HFpEF (EF above 50%) per echocardiogram.	6 months	Anamnestic investigation, clinical assessment, echocar-diography, plasma samples, telephone calls.	ASE
Polat et al., 2016	HFpEF as defined by (1) history of symptoms correspondant to NYHA class II or III, (2) LVEF >50% and LVEDVI <97 ml/m2, (3) LV diastolic dysfunction per echocardiogram	N/A	Clinical assessment, echo-cardiography, serum sam-ples.	ESC, NYHA
de Boer et al., 2018	Heart failure as determined through signs and symptoms. Incident HF event as categorized by HFpEF (LVEF≥50%), HFrEF (LVEF, <50%), or unclassified.	144 months	Clinical assessment, echo-cardiography, medical rec-ords, plasma samples.	CHS: CHS criteria, ICD-9 codes 427-429.9. FHS: Framingham criteria. MESA: Identified from records. PREVEND: ESC
Wu et al., 2018	HFpEF as defined by (1) HF defined by Framing-ham criteria and normal systolic function (ejection fraction ≥ 50%), (2) echocardiographic evidence	N/A	Cardiac magnetic resonance imaging, doppler imaging, echocardiography, plasma	ESC, Framingham crite-ria, NYHA

	of LV diastolic dysfunction.		samples.	
Cui et al., 2018	HF episodes as determined by cardiologists based on biomarkers concentrations and validated according to ESC.	12 months	Clinical assessment, echocardiography, medical records, plasma and serum samples, telephone interviews.	ESC, NYHA
Ansari et al., 2018	HFpEF as defined by the CIBER study. Grade of diastolic dysfunction per ASE/EACVI Guidelines.	12 months	Echocardiography, medical records, blood samples, telephone interviews.	ASE/EACVI, NYHA
Lebedev et al., 2020	HFpEF as defined by ESC 2016	N/A	Echocardiography, medical records, serum samples.	ESC, NYHA
Merino-Merino et al., 2020	HF as diagnosed by a cardiologist. HFmrEF and HFpEF as diagnosed by LVEF between 40-49.9% versus >50%.	N/A	Echocardiography, medical records, serum samples.	Internal Criteria
Pecherina et al., 2020	HFpEF as defined by (1) no clinical signs of HF (Killip class I) and (2) LVEF ≥ 40%. HFrEF as defined by HF signs (Killip class to II-IV) and LVEF <40%.	0.5 months	Echocardiography, medical records, serum samples.	ESC, Killip class
Mitic et al., 2020	HF as defined by (1) symptoms of HF, BNP plasma concentration (> 35 pg/mL) and (2) relevant structural heart changes (LV mass index, LVMI ≥ 115 g/m2 for males and ≥ 95 g/m2 for females or L atrial dilation ≥ 40 mm) and/or diastolic abnormality (E/A ratio < 0.75 or ≥ 1.5).	N/A	Blood samples, clinical assessment, electrocardiography, echocardiography, medical records.	ESC, NYHA
Kanukurti et al., 2020	HF as defined by clinical symptoms and HFpEF as defined by EF≥ 50% per echocardiogram.	N/A	Echocardiography, medical records, plasma samples.	Internal Criteria
Watson et al., 2021	HFpEF diagnosis as confirmed by (1) new onset HF symptoms (2) LVEF>50% and (3) elevated BNP (>100 pg/ml,), disastolic dysfunction of the LV per Doppler-echocardiogram.	N/A	Clinical assessment, echocardiography, medical records, phlebotomy, serum samples.	ESC, NYHA
Trippel et al., 2021	HFpEF as defined by presence of signs and symptoms of (1) HF, ≥2 Framingham criteria for HF, (2) a preserved LVEF > 50%, and (3) echocardiographic findings of LV diastolic dysfunction. The diagnosis was established when left atrial volume index >34 mL/m^2, or LV mass index (LVMI) ≥115 g/ m^2 for men and ≥95 g/ m^2 for women, or E/e′ ≥ 13, or mean e′ septal and lateral wall <9 cm/s.	120 months	Clinical assessment, echocardiography, medical records, plasma samples.	ESC, Framingham criteria

Acute Heart Failure (AHF); American Society of Echocardiography (ASE); B-type natriuretic peptide (BNP); Cardiovascular Imaging and Biomarker Analyses (CIBER); Cardiac Magnetic Resonance Imaging (CMRI); European Association of Cardiovascular Imaging (EACVI); European Society of Cardiology (ESC); Heart Failure (HF); Heart Failure with Preserved Ejection Fraction (HFpEF); Heart Failure with Reduced Ejection Fraction (HFrEF); Left Ventricular (LV); Left Ventricular End Diastolic Volume Index (LVEDVI); Left Ventricular Ejection Fraction (LVEF); New York Heart Association (NYHA).

Table S2. Study assays.

Authors	Sample Type	Storage Duration (Years)	Storage Temperature (°C)	Galectin Measurement Method*	Assay Manufacturer
Yin et al., 2014	Plasma	N/A	-70	ELISA	BG Medicine, Waltham, MA
Wu et al., 2015	Plasma	N/A	-80	ELISA	R&D Systems, Minneapolis, MN
Yu et al., 2015	Plasma	N/A	-80	ELISA	BG Medicine, Waltham, MA

Edelmann et al., 2015	Plasma	N/A	-80	ELISA	BG Medicine, Waltham, MA
Berezin et al., 2016	Plasma	N/A	-70	ELISA	BG Medicine, Germany
Beltrami et al., 2016	Plasma	24 hours	N/A	ELISA	eBioscience, CA, USA
Polat et al., 2016	Serum	N/A	-80	ELISA	eBioscience, CA, USA
de Boer et al., 2018	Blood samples	N/A	N/A	ELISA	BG Medicine, Waltham, MA
Wu et al., 2018	Plasma	N/A	-80	ELISA	R&D Systems, Minneapolis, MN
Cui et al., 2018	Venous Blood	N/A	-80	Human Gal-3 Assay Kit	Immuno-Biological Laboratories Co., Japan
Ansari et al., 2018	Serum	N/A	-80	Architect System	Abbott Laboratories
Lebedev et al., 2020	Serum	N/A	-80	ELISA	R&D systems, Inc. Minneapolis, MN
Merino-Merino et al., 2020	Blood samples	N/A	N/A	Architect System	Abbott Laboratories
Pecherina et al., 2020	Plasma	N/A	N/A	ELISA	Thermo Fisher Scientific, Waltham, MA, USA
Mitic et al., 2020	Plasma	N/A	-80	Quantikine USA Kit	R&D Systems, Inc. Minneapolis, MN, USA
Kanukurti et al., 2020	Serum	N/A	-40	ELISA	Elabscience Human GAL3 ELISA Kit, Houston, United States
Watson et al., 2021	Serum	N/A	-80	Architect System	Abbott Laboratories
Trippel et al., 2021	Venous Blood	N/A	-80	ELISA	BG Medicine, Inc., Waltham, MA, USA

*An enzyme-linked immunosorbent assay (ELISA) is a highly sensitive test used to detect and quantify substances including antibodies, antigens, proteins, and hormones in biological samples(36).

Table S3. Minimally Adjust HR when provided.

Study Authors	Minimally Adjusted HR [95% CI] (Covariates)
Yin et al., 2014	N/A
Wu et al., 2015	N/A
Yu et al., 2015	N/A
Edelmann et al., 2015	N/A
Berezin et al., 2016	N/A
Beltrami et al., 2016	HR: 23.98 [3.03–89.45]; p = 0.001
Polat et al., 2016	N/A
de Boer et al., 2018	N/A
Wu et al., 2018	N/A
Cui et al., 2018	N/A
Ansari et al., 2018	N/A
Lebedev et al., 2020	N/A
Merino-Merino et al., 2020	N/A
Pecherina et al., 2020	N/A
Mitic et al., 2020	N/A

Kanukurti et al., 2020	N/A
Watson et al., 2021	1.15 [1.03-1.28]
Trippel et al., 2021	N/A

Table S4. Limitations of included studies, as reported in the body of the text (and edited for concision) and as identified by authors.

Study Authors	Study Limitations
Yin et al., 2014	Covariates in adjusted model were not stated, HR nor OR were used as the statistical test, retrospective, serial galectin-3 measurements lacking, small percentage of female participants, small sample size
Wu et al., 2015	CMRI was not performed in all patients, diabetes not recorded as a comorbitity, HR nor OR were used as the statistical test, PASP data lacking, recruitment did not account for DHF as a distinct syndrome, retrospective, serial galectin-3 measurements lacking
Yu et al., 2015	Diabetes not recorded as a comorbitity, further adjusted HR or stated covariates used in adjusted model lacking, HF study population has CHD which limits generalizability, inclusion of biomarkers with potential prognostic value in HF lacking, serial galectin-3 measurements lacking, small HFpEF sample size
Edelmann et al., 2015	Ambiguous whether galectin-3 increased first or the clinical event occurred first, Clinical events driven by HF hospitalization, did not adjust for other biomarkers, low number of clinical events, Post-hoc analysis, small number of patients with an increased galectin-3
Berezin et al., 2016	HD-FACS methodology is not standardized, lack of serial galectin-3 measurements., retrospective, small sample size
Beltrami et al., 2016	Diastolic filling measurement not compared with invasive analysis, did not adjust for other biomarkers, follow-up period only 6 months, observational, retrospective, serial biomarker measurements lacking, small sample size
Polat et al., 2016	Covariates lacing in further adjusted model, follow-up lacking, HR nor OR were used as the statistical test, retrospective, serial galectin-3 measurements lacking, small sample size
de Boer et al., 2018	30% of HF cases were unclassified, lack of other biomarkers as covariates, LVEF point to distinguish HFrEF and HFpEF is debated, only some cohorts had all biomarkers, serial biomarker measurement lacking, variable durations between enrollment and incident HF event
Wu et al., 2018	Correlative findings only, cross-sectional, diabetes not recorded as a comorbitity, direct tissue biomarker expression lacking, follow-up lacking, serial biomarker measurement lacking, small sample size
Cui et al., 2018	Galectin-3 cut-off value to distinguish HFpEF from controls differed from results of the ALDO-DHF study, galectin-3 is not a cardiac-specific biomarker, retrospective, serial biomarker measurement lacking, small sample size
Ansari et al., 2018	Bias in patient selection towards individuals with symptoms, participants were all sampled from a population undergoing routine echocardiography at an outpatient department in various stages of HF, prospective, serial galectin-3 measurement lacking, small sample of patients with severe diastolic dysfunction, small sample size, three different examiners carried out echocardiographic evaluation
Lebedev et al., 2020	Covariates for adjusted model lacking, follow-up lacking, HR nor OR were used as the statistical test, MMP-9 and TIMP-1 measurements in serum can overestimate concentrations compared with measurments from plasma, retrospective, severe HF (III-IV functional class NYHA) and HFrEF patients were not included, small sample size
Merino-Merino et al., 2020	Follow-up lacking, HR nor OR were used as the statistical test, impact of the biomarkers' levels on mortality or hospitalization was not evaluated, low percentage of female participants, LV mass not accounted for, patients with persistent symptomatic AF were the only ones included, retrospective, serial biomarker measurement lacking
Pecherina et al., 2020	Covariates for adjusted model lacking, HR nor OR were used as the statistical test, low percentage of female participants, risk factors and treatment variation, small sample size
Mitic et al., 2020	Age lacking, diabetes not recorded as a comorbitity in controls, follow-up lacking, HR nor OR were used as the statistical test, retrospective, serial biomarker measurement lacking
Kanukurti et al., 2020	Randomization and follow-up lacking, cross-sectional, diabetes not recorded as a comorbitity, HR nor OR were used as the statistical test, serial biomarker measurement lacking, single center data source, small sample size
Watson et al., 2021	Diabetes not recorded as a comorbitity, lack of follow-up, population diversity lacking, retrospective, serial biomarker measurement lacking, Small sample size
Trippel et al., 2021	Changes in cardiovascular medications, invasive hemodynamics lacking in diagnosis ascertainment, lack of follow-up, lack of

	population diversity, Neurohormonal activation not assessed, potential effect of diuretic treatment and other pharmaceutical therapy, serial galectin-3 measurement lacking, small sample size and event rate, temporal dispersion of follow-up echocardiography

Atrial fibrillation (AF); Aldosterone Receptor Blockade in Diastolic Heart Failure (ALDO-DHF); Cardiac Magnetic Resonance Imaging (CMRI); Congenital Heart Defects (CHD); Diastolic Heart Failure (DHF); Hazard Ratio (HR); Heart Failure (HF); Heart Failure with Preserved Ejection Fraction (HFpEF); Heart Failure with Reduced Ejection Fraction (HFrEF); High Definition Fluorescence Activated Cell Sorting (HD-FACS); Left Ventricular Ejection Fraction (LVEF); Matrix Metalloproteinase-9 (MMP-9); Odds Ratio (OR); Pulmonary Artery Systolic Pressure (PASP); Tissue Inhibitor Metalloproteinase-1 (TIMP-1).
